# Evaluating the Home-Based Intervention Study (HIS-UK) to Improve Condom Use Skills and Experience: A Qualitative Study

**DOI:** 10.1080/19317611.2024.2382246

**Published:** 2024-08-01

**Authors:** Rowena Bedford, Lauren Towler, Nicole Stone, Cynthia A. Graham

**Affiliations:** a Centre for Sexual Health Research, Department of Psychology, University of Southampton, Southampton, UK; b Department of Gender Studies, The Kinsey Institute, Indiana University, Bloomington, IN

**Keywords:** Condom use, sexual health, intervention, young men

## Abstract

**Objectives:**

We explored participants’ experiences in a randomized controlled trial that evaluated the Home-Based Intervention Strategy (HIS-UK) to improve male condom use skills/experiences.

**Methods:**

25 men (18–25 years) who had reached the first 6 months of the intervention were interviewed. We used a codebook approach to thematic analysis to analyze the data.

**Results:**

Four themes were identified: Perceived benefits of online versus in-clinic recruitment; Perceptions of the educational content; Benefits of the condom kit; Acceptability of condom testing and monthly questionnaire.

**Conclusion:**

Participants valued the intervention, especially the condom kit that provided a range of different condoms and lubricants.

## Introduction

Chlamydia is the most prevalent sexually transmitted infection (STI) diagnosed in the United Kingdom (U.K.) and rates continue to increase, particularly among young people (Health Security Agency, 2023). In 2022, 37,404 young men aged 15–24 tested positive for Chlamydia, compared to 29,317 in 2021 (Health Security Agency, 2023). There has also been a noticeable shift in how young people access Chlamydia tests, particularly since the COVID-19 pandemic, with many using online STI testing services, removing the need for face-to face contact in a clinic (Lewis et al., 2021). It is well documented that if used correctly and consistently male condoms are 98% effective at protecting against unplanned pregnancy and most STIs, including Chlamydia (World Health Organization, 2023a), but despite the high efficacy, condoms are often not used correctly or consistently and are often perceived negatively by both men (Crosby et al., 2007) and women (Duerr et al., 2011).

Condom errors and problems have been documented as frequent reasons for inconsistent or incomplete use, as well as failure. Errors include not leaving enough space at the tip, not using water-based lubricant, incorrect withdrawal (Sanders et al., 2012), and not checking for damage and contact with sharp objects (González-Hernández et al., 2020). Reported problems include slippage, breakage, and condom-associated erection difficulties (Sanders et al., 2012).

Major barriers to condom use include poor fit (size and shape) (Crosby et al., 2010) and feel (physical sensations) (Crosby et al., 2013), contributing to reduced pleasure. Discomfort is closely linked to tightly fitting condoms and dryness, and associated with reduced sensation, breakage, and incomplete use (Crosby et al., 2005). Fit issues are related to condoms being too long/short and not rolling down fully to cover the penis, leading to condom slippage (Reece et al., 2010). Feel problems often relate to the reported lack of “naturalness”, with the condom reducing sensitivity and inhibiting orgasm (Davis et al., 2014). Other barriers relate to the notion that condoms can disrupt intimacy and spontaneity (Braun, 2013). All such factors can increase the likelihood of condom discontinuation and avoidance. The CONUNDRUM study of young people aged 16–24 living in Scotland (Lewis et al., 2021) reported a reluctance by users to discuss personal requirements such as condom size and material. This may be due to a fear of being judged about condom size or sexual activity and might be particularly salient for sexual and gender minority youth. This discourages a healthy and educated conversation about condom practices with health care professionals.

With a reliance by young people on seeking sexual health services online, it is important to also deliver educational resources through a similar route. Digital health studies have been shown to have a positive impact on sexual health by improving self-efficacy, sexual behavior, and intention to use or carry condoms (Bailey et al., 2010). However, there are barriers to engaging with sexual health online content. These may range from wariness to engage with visual and auditory content, lack of trust for online sources, and fear of observation e.g., from parents for young people living at home (Patterson et al., 2019).

The Home-Based Intervention Strategy (HIS-UK) is a brief behavioral intervention targeted to young men (aged 16–25) to improve condom use skills and enhance pleasurable condom use. HIS-UK is an adaptation of the Kinsey Institute Homework Intervention Strategy (KIHIS), developed in the U.S (Emetu et al., 2014; Gesselman et al., 2020; Milhausen et al., 2011). The intervention aims to increase condom use and enjoyment by enhancing fit and feel through the practice of applying, using and removing a range of different condoms and lubricants alone in a “low pressure” environment (Milhausen et al., 2011). KIHIS was adapted for use in the U.K., with consultation from both young men and sexual health professionals. A feasibility study was then conducted to inform the design, methodology, and data collection to evaluate HIS-UK in a randomized controlled trial (RCT) (Stone et al., 2018). Two HIS-UK education and delivery models were developed: (1) face-to-face by a health professional (proHIS) and (2) a digital interactive website (eHIS). Findings indicated the intervention was acceptable to young men and health professionals and the outcome measures appropriate (Stone et al., 2018).

Following this, the RCT with three arms – proHIS, eHIS, and standard care – was implemented. The primary health outcome of the trial was Chlamydia test positivity at six months and at twelve months post-enrolment. The effectiveness of HIS-UK at reducing new Chlamydia infection compared to standard NHS condom delivery was assessed at three time points (baseline, six, and twelve months) using in clinic or at home testing kits. Men who reported sex with other men at baseline were triple tested (oral, anal, and urine swab), with all other participants providing a single urine test. Secondary outcomes collected via online questionnaires included monthly changes in behavioral measures related to condom attitudes, barriers, errors, and problems. Measures included the Condom Barriers Scale (St. Lawrence et al., 1999), the Condom Use Errors and Problems Survey (Crosby et al., 2020), the Condom Use Self-Efficacy Scale (Charnigo et al., 2010), and the Multidimensional Condom Attitude Scale (Helweg-Larsen & Collins, 1994). Other secondary outcome measures, collected from baseline through to month twelve, included a sexual behavior and contraceptive use survey (contraception use, sexual partner history, relationship status/type, frequency of intercourse) and the cost effectiveness of eHIS and proHIS compared to standard care (NHS condom delivery).

In the study HIS-UK arm participants were provided with a condom kit containing a wide range of condoms of different brands, shapes and sizes, lubricants (24 condoms and 12 lubricants in total), a condom guide, and a condom “fit kit”. Supplying individuals with a large array of different condoms is a valuable teaching tool to educate about condom fit-and-feel (Stone et al., 2018). Participants were asked to complete a two-week self-practice testing period with the kit, focusing on pleasurable sensations, and to rate the condoms in terms of fit-and-feel after each condom practice session on an online form. Participants had further opportunities to order more supplies of the condoms and lubricants of their choice each month for up to twelve months. As a thank you for participation, upon study completion participants were either provided a further supply of condoms and lubricants of their choice (study arm) or a condom kit (standard care arm), with the eHIS delivery link also provided. At completion participants were provided with up to £50 worth of electronic distributed vouchers at different time points: £10 at three months, £15 at six months, and £25 at month twelve.

### Current study

To assess participants’ experiences in the study, qualitative semi-structured interviews were carried out between March 2022 and May 2023 with participants who had reached the six-month time point of the study. Interviews assessed four main areas: (1) study acceptability, (2) adherence, (3) possible issues with the protocol, and (4) perceived benefits and areas for improvement. This paper reports on the qualitative data from this sub-study.

Research questions were: Were participants study expectations met? Was the educational content informative? Was the consultation process straightforward? How did participants perceive the materials provided? What did participants see were the main benefits of participating? Were there areas for improvement in the study procedure or the intervention?

## Materials and methods

### Participants

Inclusion criteria included men and people with attributes of a biological male (i.e., a penis) aged 16–25 years based in England, self-reported to be at risk of STIs through condom use errors (i.e., breakage/slippage) or condomless penile-vaginal/penile-anal with casual/non-regular or new partners in the last three months. All participants gave informed consent and expressed willingness to be committed to the 12 months study duration.

Participants were recruited via two pathways: the sexual health clinic route (clinic team referral) or the community route (self-referral). Clinic referral involved eight participating National Health Service (NHS) Trusts across England. The COVID-19 pandemic, which started the same month our recruitment opened, presented a serious challenge for the study. Recruitment figures were impacted due to clinic staff illness, clinic closures, and the transfer of clinic staff to vaccine trials.

The introduction of the community (self-referral) route was invaluable to side-step the clinic process and boost recruitment. Study delivery was conducted by the HIS-UK research team rather than in-clinic staff. Condom kit delivery, the Chlamydia testing kit and receipt of results were coordinated with an external laboratory and exclusively by the research team. This route introduced Participant Identification Centers (PICs) in the form of General Practitioners surgeries to access participants outside the clinic catchment area. PICs sent a text message to eligible young men with a link to the study sign-up page.

### Measures and procedure

At the six-month follow-up period, the participants were asked whether they would be willing to be contacted (via Lifeguide^1^) by the research team to participate in a short interview remotely via Teams (either telephone or face-to-face) about their involvement in the study. The research team were notified of interested participants via email and participants were purposively sampled based on characteristics such as referral route and trial arm to ensure a range of participants were represented. Selected participants were sent an email invitation including a Calendly link to book a suitable time slot for the interview. The interview took approximately 20 minutes.

The first and second authors conducted all the one-to-one interviews. At interview commencement, participant attendance was logged by submitting details (i.e., ID, email) for financial reimbursement. The aims of the interview were briefly described verbatim, along with confidentiality, and the right to withdraw. The interviewers ticked an online box after verbally establishing the participant’s consent. All interviews were completed via Teams video conferencing.

The following brief demographic information was obtained: age, ethnicity, highest level of education completed, student/non-student status, and STI diagnosis ever (see Table 1 for response options for each of these variables).

**Table 1.** Demographic Characteristics of Sample (*N* = 25).

	n	%
Ethnicity		
White (British/Irish/Other)	23	92
Black (Caribbean/African/Mixed)	1	4
South Asian	1	4
Highest level of education completed		
None from list	1	4
Secondary school	11	44
Business/technology professional qualification	12	
Bachelor’s degree	6	24
Master’s degree	4	16
Student status		
Full-time student	16	64
Non-student	9	36
STI diagnosis ever		
Yes	9	36
No	16	64

The semi-structured interview guide was based on the Process-Evaluation framework from Saunders et al. (2005), a systematic and comprehensive approach for developing a process-evaluation plan to assess the implementation of health promotion interventions. Suggested plan elements include fidelity, dose (delivered and received), reach, recruitment, and context. The interview guide is presented in Appendix A. All interviews were audio-recorded via Teams, and the interview finished by debriefing participants and providing them the opportunity to ask questions.

Payment consisted of a £10 Amazon voucher for participants’ time. Approval for the study was obtained from the NHS Ethics Approval was granted by the South Central - Oxford B Research Ethics Committee [IRAS ID: 255684 (HRA Approval 19.11.19].

### Data analysis

A codebook approach to Thematic Analysis (TA) was used to analyze the data (Braun & Clarke, 2022). This analysis defines codes and themes by including a definition and exclusion for each code, as well as examples for each code (Roberts et al., 2019). The codebook approach was particularly valuable in this study as it provided an efficient way to help identify patterns in the dataset and explore the data, as well as a useful layout for study authors to review the data. Audio-recordings were transcribed verbatim, and data anonymized, a process completed independently by the first two authors.

Transcripts were imported into NVivo. The first author created a simple initial codebook of deductive codes and categories based on the study aims (e.g., how participants felt about the condom kit, specific intervention elements etc.) and began (after initial familiarisation) coding the data deductively and also identifying inductive codes that fell within the overarching research questions. The coding was done by unit of meaning. After this stage, the code list was reviewed and the codebook updated to reflect the new categories and preliminary candidate themes, which was reviewed by the second author. Any suggested changes were checked against the coded data, and some themes/categories were split or merged.

The study aims were to inform the Process-Evaluation framework (Saunders et al., 2005); therefore, the codebook encompassed acceptability, protocol adherence, perceived benefits, and possible study improvements. Concepts were then imported into Microsoft Word with codes discussed regularly among all the authors to create the final thematic structure (see Supplementary Material).

## Results

Twenty-five semi-structured interviews with participants aged 18–25 years (mean age 22 years) were conducted, seventeen from the HIS-UK arm and eight from the standard care arm (see Table 1 for demographic characteristics of the sample). Eleven interviewees had been recruited to the study via the community route (proHIS n = 4, eHIS n = 6, standard care n = 1) and 14 had been recruited in clinic (proHIS n = 4, eHIS n = 3, standard care n = 7). During the interview process, six participants were “no-shows”; however, three were subsequently rescheduled and completed interviews. Due to how recruitment was done, reasons for declining participation were not recorded.

Overall, four main themes were identified: (1) Perceived benefits of online versus in-clinic recruitment; (2) Perceptions of the educational content; (3) Benefits of the kit: Novelty, variety, and convenience; and (4) Acceptability of condom practice sessions and monthly questionnaires.

### Perceived benefits of online versus in-clinic recruitment

Participants identified many different considerations when weighing up their preferences for online participation versus clinic attendance. Overall, most participants expressed a preference for the online route. This was largely due to the convenience of online communication, particularly since the advent of COVID-19 and Mpox,^2^ where securing a clinic appointment was more challenging. Participants could explore the study within the comfort of their own homes, in their own time and at their own pace. This format was particularly advantageous for participants who usually experienced discomfort or avoidance at the prospect of attending a clinic. Offering the opportunity to participate remotely enabled us to target hard-to-reach groups within the community such as Black and minority ethnic (BME), individuals with disabilities, and travelers (Flanagan & Hancock, 2010). One participant highlighted the obstacles faced in communicating about topics related to sexual health:

“Sexual health is quite a funny one, it’s quite a silent mentality towards it. So, let’s talk about other people’s opinions. I reckon some people would prefer it online” (P^3^ 25, aged 21 years, proHIS).

However, four participants considered this sense of distance from the research team would lead to disengagement within the study. The home environment could also be a distraction and reduce the likelihood of long-term retention of content received.

“I'm more likely again to retain the information because someone told me rather than it being at home, being distracted” (P 23, aged 25 years, eHIS).

There was also a sense of distrust with centering the interaction online rather than face-to-face. This notion was highlighted in a study by Freeman et al. (2020), where young people worried about encountering unreliable or inconsistent information with the vast volume of information available, although this did not stop them from online searching. In the current study, two participants viewed seeing a healthcare professional in person as more reliable, particularly to reduce the risk of misdiagnosis and identification of any possible health concerns.

“I usually prefer going into the clinic because I’ve had stuff in the past. I’ve had a UTI and thought it was a STI” (P 10, aged 25 years, standard care).

For two participants, the online STI testing procedures compared to clinic testing were also considered more appealing compared to standard care. The introduction of a pipette in the study test kit for urine samples was regarded as aiding sampling efficiency, a feature missing from standard care kits for participants.

“…the sexual health clinic they just give you a vial that you fill up… but they don’t provide a pipette…The pipette is easier to get direct measurements” (P 21, aged 25 years, proHIS).

In sum, the online recruitment route and study procedures were seen as a valuable resource for convenience and as a way to target those who would not usually attend a sexual health clinic. However, the in-clinic route may have better long-term benefits for information retention. Moreover, considering participants’ concern of possible misdiagnosis, it may be worthwhile for future research to investigate ways to manage this issue. Turner et al. (2022), for example, provided online guidance for clinicians and practice managers listing possible unintended consequences of online consultations (including miscommunication) and methods to mitigate these, such as providing clear instructions for what is deemed appropriate for online consultations.

### Perceptions of the educational content

Participants’ perceptions about their overall experiences with the study were impacted by study arm allocation, with more positive experiences generally reported by the proHIS participants. However, while six participants acquired newfound skills or consolidated prior knowledge, others described limited benefit. The usefulness of the study material was largely dependent on whether participants had experienced comprehensive sexual health advice prior to the study. Those participants tended to be less engaged and therefore skipped sections on the website. As one participant reported after viewing the animations within eHIS:

“…I think they’re sort of animations that I've seen quite a lot anyway that you get when you’re younger and in sex eds. So don’t think if I'm being completely honest, I paid too much attention to them” (P 23, aged 25 years, eHIS).

However, three participants described learning additional skills beyond their school sex education classes. The intervention emphasized the valuable role of sexual pleasure in condom use, a topic unfamiliar to most participants. This may be due to pleasure being simply unmentioned, with instead a greater focus on possible negative outcomes within the school curriculum such as the avoidance of STIs, unplanned pregnancy, and coercion from peers (Ingham, 2005). Through introducing the importance of pleasurable condom use, this challenged seven participants’ previously held negative views of condoms.

“I never knew how important heat transfer and stuff was…I always just thought I absolutely despise condoms” (P 21, aged 25 years, proHIS).

Moreover, three participants saw the study as a useful opportunity to refresh current knowledge. Of particular benefit was the proHIS condom demonstration. This may have been aided further by the face-to-face online component where participants could learn in a safe environment and freely ask questions, away from a group setting (such as a school sexual health class).

“…it’s good to see a good demonstration of how you are meant to do it. And I can still picture it in my head now” (P 25, aged 21 years, proHIS).

This was also the case for four standard care participants who found the website links to sexual health advice helpful, although not necessarily providing new information.

“There were many different sources of information about different websites…they all seem really good, very useful, but I think I just had quite good understanding already” (P 8, aged 21 years, standard care).

Standard care participants also described the study as an opportunity to reflect on their current condom use behaviors and attitudes. For six participants, this promoted condom awareness and condom use.

“I guess because the study maybe reminded me to do it safely” (P 2, aged 19 years, standard care).

In general, standard care (in clinic) condom education and training was perceived as limited. Participants reported that they were only provided with a small supply of condoms (typically of one brand) and occasionally some lubricants.

“…they gave me some [lubricants] in the bag, but not really much about them” (P 10, aged 25 years, standard care).

However, one participant did describe the provision of condoms in waiting areas and consultation rooms across clinic settings.

“Every clinic I've been to like the waiting room and every consultation room will just have like a big ball of them [condoms] or like a bag of them”. (P 4, aged 20 years, standard care).

Thus, views about the study arms and educational content were mixed. Five participants embraced the new learning opportunity, others were less committed when the information was already believed to be common sense or already known. Future interventions could focus on participants who consider such material to be like school sex education classes, and emphasize how the study content goes above and beyond usual sex education, for example, about the benefit of lubricants in condom use both for individuals and for their partner(s).

### Benefits of the kit: novelty, variety, and convenience

The condom kit (see Appendix B) was the standout benefit for participants, with the number and variety of condoms and lubricants provided exceeding expectations. With the intervention encouraging exploration and the element of choice, participants were able to compare brands, shapes, and sizes. This helped to identify the best condom for fit-and-feel and in some cases change preexisting negative views about condoms.

“I would normally just go out and buy like normal condoms like regular thickness and stuff like that… that’s probably why I dislike them” (P 21, aged 25 years, proHIS).

Participants were also surprised by the novelty of the shape and size condom fit kits.^4^ This ruler-like tool aimed to give participants a visual understanding of how their penis size/shape corresponded to different condoms. The kit challenged previously held condom size beliefs, directing participants to better suited condoms.

“I thought they were mind blowing…you kind of assume that you fit into an average. And then like when it came to it… I clearly need to be looking at different options” (P 22, aged 25 years, eHIS).

The positive views of the kit extended beyond the study practice sessions, with participants using the bag for traveling and praising its accessibility and solution when not having a condom present. The condom kit may therefore further promote keeping a supply of condoms both at home and away from home.

“If I’m going over to someone’s house, I put it in my bag and have access to it. People have commented that’s a really good idea” (P 21, aged 25 years, proHIS).

Moreover, five participants acknowledged the importance of lubricants and the difference between water and silicone-based and identifying their favored type. Lubricants and their use in the shower were found to be particularly insightful to participants, namely establishing lubricants that were waterproof and non-greasy. However, it was apparent that some participants were still confused between the two types of lubricants:

“I feel like water-based ones can break condoms, can’t they? Or is that the other way around?” (P 22, aged 25 years, eHIS).

This confusion may be largely due to certain participants (n = 3) admitting that they skipped the lubricant section due to having no interest in their use.

“I probably skipped the bit on how to use it because I’m not interested in lubricants” (P 17, aged 20 years, eHIS).

This suggests that participants didn’t completely recognize the importance of lubricants or how they could be beneficial for pleasure.

Other potential issues were related to the two-week time allocated for testing. Being overwhelmed by the number of condoms and having a lack of time/energy to rate their experiences with them may have resulted in missed feedback. However, when asked if this timeframe should be lengthened, most noted two weeks was sufficient.

“There were a lot of them which I didn’t try out because I just didn’t have the time or the convenience or the mood” (P 16, aged 22, proHIS).

Finally, through replacing the typical condom retail boxes in favor of a ‘washbag’ style kit, two participants were unclear on the features of each condom supplied. This was particularly the case for more ambiguously named condoms. One participant suggested providing a booklet in the kit to describe each condom.

“You’re not sure which each one is meant to do…… The magnum could be it tasted like ice-cream rather than extra-large” (P 16, aged 22, proHIS).

Overall, the kit was the defining feature of the study and provided participants the unique opportunity for condom and lubricant exploration. The supply was also a distinct contrast to what was provided in clinic settings that usually offered a limited number and variety of condoms.

“I think it had about six of a few different types, so there’s some non-latex ones and then some different sized ones…I think they were the Pasante ones” (P 13, aged 20 years, standard care).

### Acceptability of condom practice sessions and monthly questionnaires

Some participants initially wondered why they needed to try the condoms out on their own and regarded practicing with a partner as more effective. However, when realizing how many condoms they were asked to try, time constraints meant self-practice was the best option. It was also acknowledged that self-practice would be the most effective way to allow time to identify the best condom for fit-and-feel.

“I think a part of you initially think I should probably try these with a partner, but then you’re like there is no way that I'm having that much sex in such a short span” (P 22, aged 25 years, eHIS).

Other participants, however, did not see any notable differences of self-practice compared to use with a partner, primarily citing that once the skill was learnt, it doesn’t need to be practiced repeatedly to be remembered.

“I think it exaggerated the importance… once you’ve put one on correctly once it’s not a very difficult skill to remember how to do and continue to do successfully” (P 19, aged 23 years, eHIS).

One of the most notable concerns reported by participants was in relation to the monthly follow-up questionnaires, which might be the reason for some of the participant dropouts.

“I guess any potential like positive impact…was undone by the repetition and tediousness” (P 19, aged 23 years, eHIS)

This may have also impacted response quality. One participant described question familiarity and repetition resulted in responding to answers “in the middle”. To reduce the repetition, it was suggested that responses could be automatically pre-completed with the ability for participants to edit if required.

“…if there’s a question that has been filled out and answered the same way and is unlikely to change, have it pre-filled out and change it if you want to” (P 23, aged 25 years, eHIS).

Moreover, one participant who had sex with men argued that some questions were less tailored toward them. For example, the pregnancy related questions were not considered of any relevance.

“…have I gotten anyone pregnant within the last x number of months or days? Because I was only having sex with men it didn’t feel right putting not to my knowledge” (P 21, aged 25 years, proHIS).

Future researchers should consider using survey logic where questions about pregnancy are only asked for those who report female partners. To conclude, not all participants understood the importance of the self-practice period for testing and rating the condoms. The monthly questionnaires, whilst useful to track changes in behaviors and attitudes, may have been overly long and negatively impacted retention. Future interventions could therefore benefit from tailoring measures to the anticipated attention span of the participant group.

## Discussion

We aimed to understand participants’ experiences of the HIS-UK intervention and trial, examining attitudes and acceptability toward each stage from recruitment to six months. It was important to investigate whether participants enjoyed the HIS-UK program and adhered to the protocol, considered the study to be effective in bringing about attitudinal and/or behavioral change, and if they had any suggestions for possible areas for improvement.

Findings revealed reactions to the condom kit to be very positive, with the range of condoms appealing to participants who had different condom issues and requirements. The kit offered novelties, such as the condom fit kit and the ribbed and dotted condoms (aimed for pleasurable condom use). Through offering the benefit of comparison, some participants either continued with their usual condom or decided to change brands. Participants’ experiences challenged their preexisting dislike of condoms. Our findings are consistent with a previous feasibility study of the HIS-UK intervention, which indicated participants’ strong acceptance of the intervention and the directed self-practice tasks (Stone et al., 2018) and with qualitative data from the KI-HIS feasibility study (Milhausen et al., 2011).

These findings are relevant to the variables specified in the Process-Evaluation framework (Saunders et al., 2005). Participants were both actively engaged and receptive to the condom kit (exposure) as well as satisfied with the study (satisfaction). The condom kit holds promise for use in healthcare settings. Although it would incur an initial added financial expenditure, the promotion of condoms as a fun and personalized activity may lead to long-term benefits, specifically improved condom experiences, less frequent condom errors and problems, and ultimately, a reduction in STI rates.

The HIS-UK intervention emphasizes the importance of pleasure in condom use. It was apparent that many participants were not aware of methods to increase pleasure, such as through lubricant use and using condoms that fit-and-feel the best. The most frequent source of condom education and training was in school; however, sexual pleasure is very rarely included in the syllabus (Hirst, 2013). It may therefore be beneficial from an early stage of an individual’s sexual and reproductive health education journey to discuss pleasure as an additional strategy for the protection of STIs and unplanned pregnancy (Philpott et al., 2021). The importance of the promotion of pleasure in sexual health initiatives is further supported by a recent meta-analysis. Zaneva et al. (2022) demonstrated that Sexual and Reproductive Health Right (SRHR) interventions on condom use that incorporated pleasure were associated with reduced rates of HIV and STIs.

This brief intervention focusing on condom fit-and-feel and pleasure emphasized the importance of using online recruitment and delivery of the intervention as convenient, particularly to target those who would usually avoid attending clinics, and to deliver content in a safe environment for participants. However, while digital interventions are an invaluable resource in research, the home environment can also be distracting and our findings highlighted that attention spans are limited for this age group (Zapata et al., 2023). This also highlights that context cannot be ignored when examining condom use behaviors. As demonstrated in the Condom Use Experience Model (CUEs), contextual factors such as motivation are important elements that can impact condom use experience and future condom use (Sanders et al., 2012).

### Study limitations

Standard care participants were more challenging to recruit for interviews. These participants arguably were less invested, having not received the study benefits, in particular the condom kit. Community recruitment was, however, better than in clinic recruitment.

In addition, we only interviewed participants who had reached the six-month questionnaire stage. Interviews were not conducted with participants who dropped out or withdrew. While the study team sent a brief questionnaire via email to determine drop out/withdrawal reasons at the time of the request, a more detailed perspective garnered via interview is missing for these participants.

Overall, most participants used the webcam and noted this element made interviews more personable. However, two participants in the proHIS arm seem to have been deterred due to the webcam element (although this was not compulsory). An approach with greater anonymity may be more favorable. The difficulties of webcam consultations in sexual health have been emphasized by Garrett et al. (2011). In their sample of 16–24-year-olds, 85% of the sample were willing to have an in-person consultation with a doctor compared to only 29% via webcam. Participants raised concerns over consultations being recorded, saved, and then searched for online. In the current study, participant consent was obtained for interview recordings and videos were deleted post-transcription.

## Conclusion

Many participants positively received the study, and their experiences often exceeded their expectations, particularly regarding the condom kit. For the most part, they found the educational material on pleasurable condom use interesting. Perceptions of benefits were influenced by participants’ preexisting sex education. Some did not engage as well if they felt the information was already known or was “common sense”. Outlining why a topic is important and how this may impact individuals outside the study could be better explained in future studies of the intervention. To promote uptake of condoms and pleasurable condom use, it would be beneficial for clinics to introduce a wider variety of condoms and lubricants. This will encourage individuals’ exploration, finding the right condom for fit-and-feel, and enhancing positive attitudes to condoms.

## Supplementary Material

Supplemental Material

## Data Availability

The data that support the findings of this study are available in available from the corresponding author on reasonable request and will soon be openly available from the University of Southampton repository at researchdata@soton.ac.uk

## Notes

1 Lifeguide: Web based interactive digital study software developed by researchers at University of Southampton *Lifeguide Online*. Retrieved 30 October 2023 from https://www.lifeguideonline.org/.

2 Mpox (or Monkeypox) is a rare viral infection, caused by a similar virus to smallpox. Symptoms include a rash, high temperature, headache, and muscle aches. World Health Organization (2023b). *Mpox (Monkeypox)*. Retrieved 8th November 2023 from https://www.who.int/news-room/questions-and-answers/item/monkeypox.

3 P = Participant.

4 Participants were provided with two shape and size condom fit kits from Pasante and myONE. Pasante demonstrates the four sizes of condom via circular cut outs with a ruler tool for the measurements. myONE also includes a ruler, with the length letter combined with a width number to determine myONE condom size.

## Appendix

**Appendix A:** Interview guide

Topic		Sub-groups	Prompts
Enrolment	Can you remember how you found out about the study, and how you came to sign up? Did you see an advert?	In-clinic	Were the staff members knowledgeable about the study? Were you given sufficient time to read the information sheet? Was it informative? Did you feel rushed into enrolling? Did the staff answer all your questions about the study?
		Online self-referral	Did you read the participant information sheet before signing up? Was it informative? Was it clear what you would be doing?
Registration	How did you find the registration process?	In-clinic	Did you or the staff members have any problems using the tablet computer? What about the length of time it took?
		Online self-referral	Were the website instructions clear? Were the emails and instructions clear? Did you experience any problems? What about the length of time it took?
**(On-line Self-referral only)** Organizing final registration/consultation session	Was organizing the consultation with the clinical staff straightforward? Were there any delays? How was the communication?	Telephone consultation	Did you mind having a telephone consultation? Would you have preferred to see the member of staff face-to-face?
		Video Consultation	Did you mind having a telephone consultation? How did it go? Did you have any IT problems? Would you have preferred this to have been delivered in person rather than over video?
		In-clinic Consultation	Did you mind coming into the clinic to complete your registration process? Would you have preferred this to be done over the phone or by video conference?
Randomisation	Did you mind being randomly allocated to a trial group? Would you prefer to have chosen?		Remind me – what group were you allocated to?
Education/training delivery	What did you think about the information you were given about condoms?	Control	Were you given any condoms or told how to get free condoms? Was the information you were given about condoms informative? Did you learn anything new? Do you recall anything being mentioned about lubricants? Did you learn anything new about them?
		eHIS	You received information about condoms and lubes from a website – how did you find that? Was it compatible with your device? Did it function properly? What about the design? How about the video and animated clips? What about the quiz question? Was the information you were given about condoms informative? Did you learn anything new? Do you recall anything being mentioned about lubricants? Did you learn anything new about them? Would you have preferred to have had the same information about condoms and lubes given to you in a clinic setting by a health professional/nurse/doctor?
		proHIS	Was the information you were given about condoms informative? Did you learn anything new? Do you recall anything being mentioned about lubricants? Did you learn anything new about them? How did the condom demonstration go? Was it useful? Would you have preferred to have used a website to find out about the different types of condoms and lubricants?
Chlamydia screening	Did you provide samples for chlamydia testing when you first signed up? How did you provide them? (In clinic/postal kit)	In-clinic samples/ postal kit	How did you find the process? Were you informed of the results? Were you treated (if necessary)? Were you happy with what occurred?
HIS-UK Kit		eHIS/proHIS only	What did you think of the kit? Were you happy with the selection of products you were given? Were the instructions about how to test out the condoms clear? Did you mind being asked to try them out whilst alone and without a partner? Did you find that a useful exercise to do? How did you find the rating? Was it a useful exercise to do? What about ordering further supplies? Have you taken advantage of that?
Follow-up questionnaires	How have you found completing the follow-up questionnaires?		
Follow-up screening	Have you completed the 6-month chlamydia screening?	Yes	Did you experience any issues with the postal kit? Were the instructions clear? Was it easy to do?
Communication	How have you found the communication from the study team? Too much/too little/unclear		
Website	How have you found the study website? Design/compatibility with devices Any problems logging in or with access?		
Expectations	Have your expectations about the study been met? (i.e. from participate info sheet to practice)		
Perceived benefits	Do you think being involved in HIS-UK has helped you in any way? What have the benefits been? Do you think your views or attitudes to condoms and lubricants has changed in any way? Do you think your behavior has changed because of your involvement? Are you pleased you are taking part?		
Future study improvements	In your opinion, do any aspects of the study need improving or changing?		

## Appendix B

*Contents of the Home-Based Intervention Strategy (HIS-UK) condom kit*
