# Supplementary material for: Evaluating the Home-Based Intervention Study (HIS-UK) to Improve Condom Use Skills and Experience: A Qualitative Study
Source: Int J Sex Health. 2024 Aug 1;36(4):515–28. doi: 10.1080/19317611.2024.2382246 (PMC11562915; doi:10.1080/19317611.2024.2382246)
Supplement: Supplemental Material [file WIJS_A_2382246_SM8858.docx]

**Supplementary Material**

*Experiences of Participants on the Home Intervention Study ‘HIS-UK’. Thematic Organization with Definition and Example Quotes.*

| Themes | Definitions | Example Quotes |
| --- | --- | --- |
| Perceived benefits of online versus in-clinic | Participants identified different positives and negatives when weighing up their preferences to online versus clinic attendance. Overall, most participants preferred the clinic recruitment route due to the convenience and greater sense of privacy. | *“I usually prefer going into the clinic because I’ve had stuff in the past. I’ve had a UTI and thought it was a STI”* (P^^[[1]](#footnote-1)^^ 10, aged 25 years, standard care).  *“I'm more likely again to retain the information because someone told me rather than it being at home, being distracted”* (P 23, aged 25 years, eHIS).  *“Sexual health is quite a funny one, it's quite a silent mentality towards it. So, let's talk about other people's opinions. I reckon some people would prefer it online”* (P 25, aged 21 years, proHIS).  *“…the sexual health clinic they just* give *you a vial that you fill up… but they don't provide a pipette…The pipette is easier to get direct measurements”* (P 21, aged 25 years, proHIS). |
| Perceptions of the educational content | Describes some take-home messages from the intervention. Engagement appeared to be dependent on prior sexual health knowledge. | *“…I think they're sort of animations that I've seen quite a lot anyway that you get when you're younger and in sex eds. So don't think if I'm being completely honest, I paid too much attention to them”* (P 23, aged 25 years, eHIS).  *“I never knew how important heat transfer and stuff was…I always just thought I absolutely despise condoms”* (P 21, aged 25 years, proHIS).  *“…it's good to see a good demonstration [condom application] of how you are meant to do it. And I can still picture it in my head now”* (P 25, aged 21 years, proHIS).  *“…they gave me some [lubricants] in the bag, but not really much about them”* (P 10, aged 25 years, standard care).  *“I think it had about six of a few different types, so there's some non-latex ones and then some different sized ones…I think they were the Pasante ones”* (P 13, aged 20 years, standard care).  *“I guess because the study maybe reminded me to do it safely”* (P 2, aged 19 years, standard care).  *“There were many different sources of information about different websites…they all seem really good, very useful, but I think I just had quite good understanding already”* (P8, aged 21 years, standard care).  *“Every clinic I've been to like the waiting room and every consultation room will just have like a big ball of them [condoms] or like a bag of them”.* (P4, aged 20 years, standard care). |
| Benefits of the kit: Novelty, variety, and convenience | Outlines the benefits of the kit from value at offering a range of condoms to try, and the novelty of the condom shape and size guide. This theme also touches on participants perceived problems with the condom kit and feasibility in testing within the two-week self-practice period. | *“I would normally just go out and buy like normal condoms like regular thickness and stuff like that… that's probably why dislike them”* (P 21, aged 25 years, proHIS).  *“I thought they were mind blowing… you kind of assume that you fit into an average. And then like when it came to it... I clearly need to be looking at different options”* (P 22, aged 25 years, eHIS).  *“If I’m going over to someone’s house, I put it in my bag and have access to it. People have commented that’s a really good idea”* (P 21, aged 25 years, proHIS).  *“I feel like water-based ones can break condoms, can't they? Or is that the other way around?”* (P 22, aged 25 years, eHIS).  *“I probably skipped the bit on how to use it because I’m not interested in lubricants”* (p 17, aged 20 years, eHIS*).*  *“There were a lot of them which I didn’t try out because I just didn’t have the time or the convenience or the mood”* (P 16, aged 22, proHIS).  *“You’re not sure which each one is meant to do…… The magnum could be it tasted like ice-cream rather than extra-large”* (P 16, aged 22, proHIS). |
| Acceptability of condom testing and monthly questionnaires | Describes the feasibility and benefits of the condom two-week self-practice period. Participants also discuss the issues surrounding the monthly questionnaires, from repetition to the perception that questions were predominantly aimed at heterosexual men. | *“I think a part of you initially think I should probably try these with a partner, but then you're like there is no way that I'm having that much sex in such a short span”* (P 22, aged 25 years, eHIS).  *“I think it exaggerated the importance… once you've put one on correctly once it's not a very difficult skill to remember how to do and continue to do successfully”* (P 19, aged 23 years, eHIS).  *“I guess any potential like positive impact…was undone by the repetition and tediousness”* (P 19, aged 23 years, eHIS).  *“…if there's a question that has been filled out and answered the same way and is unlikely to change, have it pre-filled out and change it if you want to”* (P 23, aged 25 years, eHIS).  *“…have I gotten anyone pregnant within the last x number of months or days? Because I was only having sex with men it didn’t feel right putting not to my knowledge”* (P 21, aged 25 years, proHIS). |

1. P =Participant [↑](#footnote-ref-1)
